# Graphene-copper composite with micro-layered grains and ultrahigh strength

**DOI:** 10.1038/srep41896

**Published:** 2017-02-07

**Authors:** Lidong Wang, Ziyue Yang, Ye Cui, Bing Wei, Shichong Xu, Jie Sheng, Miao Wang, Yunpeng Zhu, Weidong Fei

**Affiliations:** 1 School of Materials Science and Engineering, Harbin Institute of Technology, Harbin, 150001, China; 2 College of Materials Science and Chemical Engineering, Harbin Engineering University, Harbin, 150001, China; 3 Department of Materials Science and Engineering, South University of Science and Technology of China, Shenzhen, 518000, China; 4 Key Laboratory of Functional Materials Physics and Chemistry of the Ministry of Education, Jilin Normal University, Siping, 136000, China; 5 School of Mechanical Engineering, Qinghai University, Xining, 810016, China

## Abstract

Graphene with ultrahigh intrinsic strength and excellent thermal physical properties has the potential to be used as the reinforcement of many kinds of composites. Here, we show that very high tensile strength can be obtained in the copper matrix composite reinforced by reduced graphene oxide (RGO) when micro-layered structure is achieved. RGO-Cu powder with micro-layered structure is fabricated from the reduction of the micro-layered graphene oxide (GO) and Cu(OH)_2_ composite sheets, and RGO-Cu composites are sintered by spark plasma sintering process. The tensile strength of the 5 vol.% RGO-Cu composite is as high as 608 MPa, which is more than three times higher than that of the Cu matrix. The apparent strengthening efficiency of RGO in the 2.5 vol.% RGO-Cu composite is as high as 110, even higher than that of carbon nanotube, multilayer graphene, carbon nano fiber and RGO in the copper matrix composites produced by conventional MLM method. The excellent tensile and compressive strengths, high hardness and good electrical conductivity are obtained simultaneously in the RGO-Cu composites. The results shown in the present study provide an effective method to design graphene based composites with layered structure and high performance.

Graphene, a two-dimensional structure of sp^2^ bonding carbon, has been intensively studied because of its extraordinary physical and mechanical properties. Its outstanding high strength and elasticity modulus^1^,^2^, remarkable electron mobility (15,000 cm^2^/V·s) and super high thermal conductivity (5,000 W·m^−1^·K^−1^) make graphene an excellent candidate as reinforcement for different kinds of composites, including polymer matrix composites^3^,^4^,^5^,^6^,^7^,^8^,^9^, metal matrix composites^10^,^11^,^12^,^13^,^14^,^15^,^16^,^17^.

Recently, copper matrix composites reinforced by nano carbon fillers, such as carbon nanotubes (CNTs), carbon nanoflakes, nano diamond and graphene, have attracted great attention since they can integrate mechanical properties (high strength and modulus) with high electrical conductivity, high thermal conductivity and low coefficient of thermal expansion^18^. However, traditional methods were not competent for the preparation of the composites reinforced by nano fillers^19^,^20^, some novel methods were explored to produce graphene reinforced copper matrix composites with designed structure and tried to clarify the role of the composite structure on the mechanical properties of the composites.

Shell nacre has a “bricks-and-mortar” structure, which comprises 95 vol.% tough aragonite “bricks” and 5 vol.% elastic organic biopolymer “mortar” in-between. This unique structure makes nacre realize both excellent strength and toughness^21^,^22^. Inspired by the structure of nacre, Xiong^23^ inserted reduced graphene oxide (RGO) into porous Cu preform and compacted into composites. This nacre-like RGO reinforced Cu matrix composite has the tensile yield strength of 233 MPa. Chen *et al*.^24^ achieved graphene *in-situ* growth on flaky Cu powders by involving ball-milling of Cu powders with PMMA as carbon source and fabricated graphene-Cu composites. The composite with 0.95 wt.% graphene has the yield strength of 144 MPa and tensile strength of 274 MPa. Nevertheless, the mechanical properties of the composites above are still far from the expectation.

Recently, Kim and his co-workers designed and produced a nanolayered graphene-Cu composite by chemical vapour deposition (CVD) and metal deposition. It is astonishing to find that the nanolayered graphene-Cu composite has an ultra-high compressive strength up to 1.5 GPa measured by nanopillar compression testing^25^. The outstanding strength of the nanolayered graphene-Cu composite was attributed to the metal-graphene layered structure and high strength of graphene. However, it should be mentioned that the technique used in the nanolayered composite is difficult to produce the composite in large scale and the nanolayered composite is very thin and anisotropic, which limits its applications.

How to produce graphene reinforced copper matrix composite with excellent mechanical properties and by a facile process is a key issue in the field of nanocomposites. Molecular level mixing (MLM) is a potential method to solve the issue, which is widely used in producing CNT^26^ and RGO reinforced copper matrix composites (RGO-Cu composites). And the yield tensile strength of 2.5 vol.% RGO-Cu composite by MLM was 284 MPa^27^. Our group introduced high-shear mixing in the process of MLM and improved the dispersity of the graphene flakes, and the compressive strength of 2.4 vol.% RGO-Cu composite reached 501 MPa^28^. These research works focus on discussing the interface strength between copper and graphene and the graphene dispersion in the composite, however, the effect of structure of the composite powders on the properties of graphene-copper composites were seldom investigated.

In this work, we fabricated a graphene-copper composite with a micro-layered structure and excellent tensile properties based on the MLM method. We found that CuO nanorods could composite with graphene oxide (GO) sheets to form a layered structure via MLM method at 45 °C with a rotor-stator mixing; this intriguing structure could be retained in the following reduction and spark plasma sintering (SPS) processes. It is believed that the micro-layered structure has a significant impact on the mechanical properties of the composites.

## Results and Discussion

### Microstructure characterizations of graphene oxide

**Figure 1:**
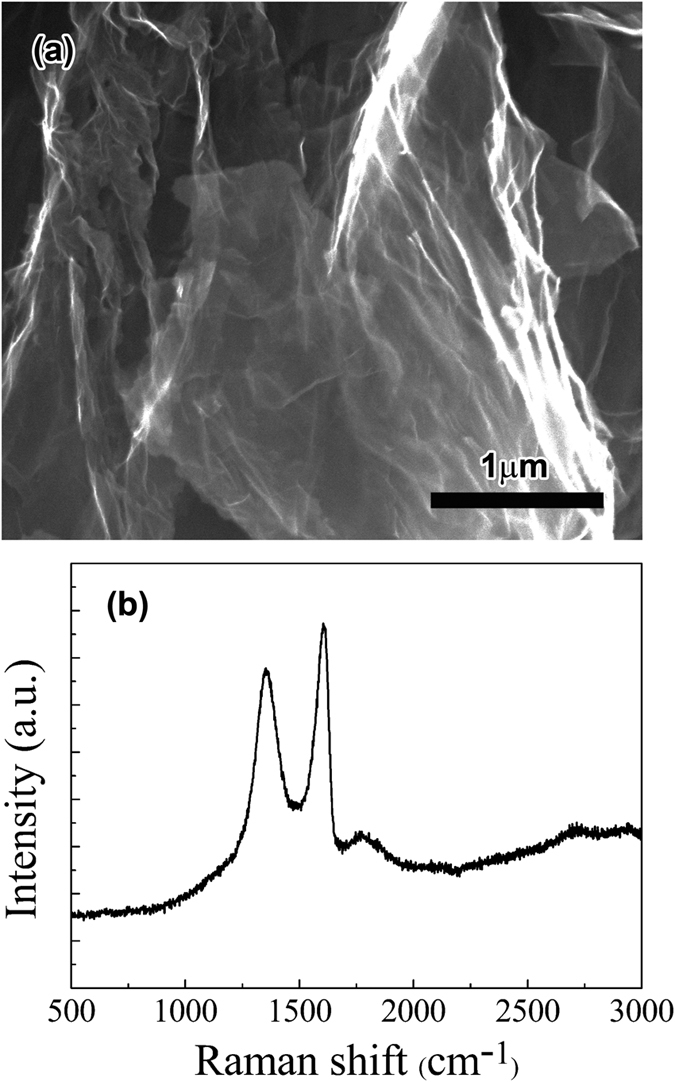
Structure characterization of GO. (**a**) SEM image of the GO flakes, (**b**) Raman spectrum of the GO flakes.

Figure 1(a) shows a scanning electronic microscopy image of GO fabricated by Hummers method. The GO flakes have a fold structure with small thickness. They are uniformly dispersed and not agglomerated. Raman spectroscopy are used to characterize the band structures of GO. As seen in Fig. 1(b), the peaks at 1353 and 1605 cm^−1^ are corresponding to D band and G band, which is consistent with the Raman spectrum of GO in previous reports^29^,^30^. D band is sensitive to the edges and defects of graphene flakes, while G band is related to the in-phase vibration of graphite lattice^31^,^32^.

### Characterizations of composite powders

**Figure 2:**
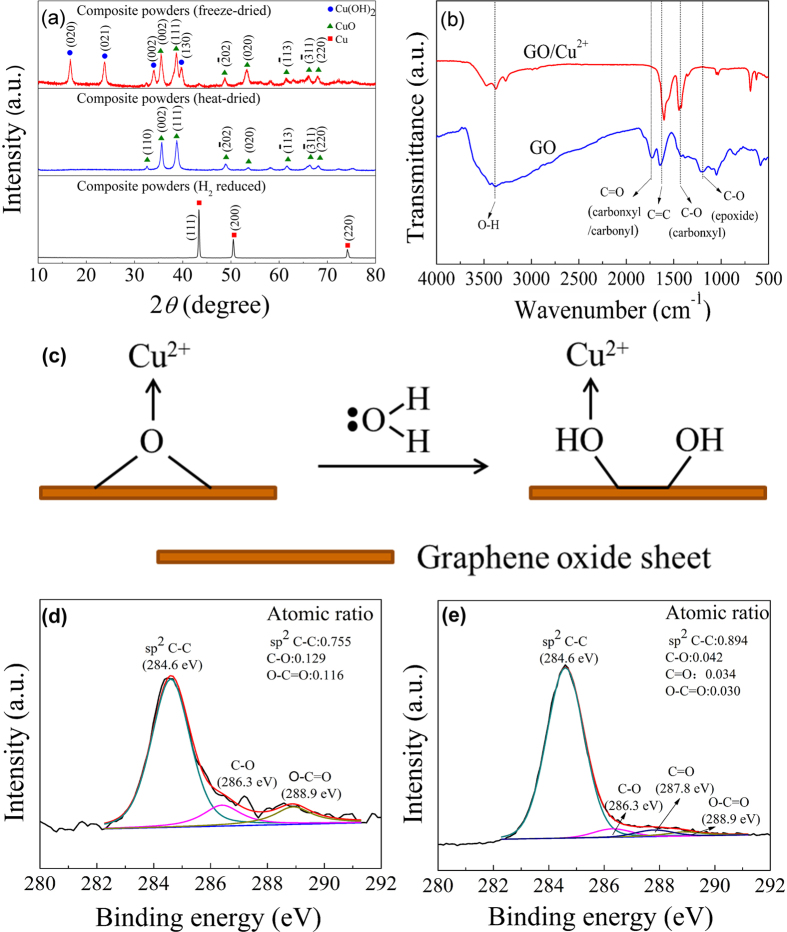
Structure characterization and phase analysis of composite powders. (**a**) XRD patterns of composite powders after freeze drying, drying in vacuum and reduction in H_2_, (**b**) FTIR spectra of GO before and after Cu^2+^ ions are added, (**c**) Proposed mechanism for the epoxide ring-opening reaction assisted by Cu^2+^ ion, (**d**) C1s XPS spectrum of GO-CuO composite powder, and (**e**) C1s XPS spectrum of RGO-Cu composite.

In order to clarify the synthesis process of the composite powder, XRD, FTIR and XPS measurements were carried out. Figure 2(a) shows the XRD patterns of the composite powders which are freeze dried, dried in vacuum at 110 °C and reduced at 400 °C by H_2_. It is noted that the freeze dried composite powder represents the original state of the composite powder produced by MLM. In the XRD pattern of freeze-dried composite powders, the diffraction peaks are assigned to the crystal planes of CuO and Cu(OH)_2_. However, in the XRD pattern of the composite powders dried in vacuum, the major diffraction peaks only match well with CuO. It suggests that Cu(OH)_2_ is formed during MLM process and gradually dehydrated into CuO during the drying process in vacuum. After reduction by H_2_, the composite powder exhibits only three characteristic peaks corresponding to Cu (PDF No. 851326), suggesting that copper oxide has been well reduced in H_2_ at 400 °C.

Figure 2(b) shows the FTIR spectra of GO before and after Cu^2+^ ions are added. The characteristic peaks of GO can be observed and indexed as the vibrations of various carbon or oxygen containing groups as shown in Fig. 2(b). It can be seen that after Cu^2+^ ions are added the C = O (carbonxyl/carbonyl) peak at ~1730 cm^−1^ is nearly invisible while the intensity of C-O (carboxyl) stretch at ~1430 cm^−1^ increased. This phenomenon has been found in refs 33 and 34 and typically interpreted as an evidence for the coordination between carboxylic acid and a divalent metal ion. In addition, the peak of C-O (epoxy) at ~1200 cm^−1^ disappears and the absorption peak of O-H (hydroxyl) becomes sharper after the Cu^2+^ ions are added, which suggests possible ring opening reactions of epoxide groups. Epoxide ring-opening reactions by Lewis acidic metal ions (such as Mg^2+^, Ca^2+^) have been reported^33^. The coordination of the epoxy O atom to the Lewis acidic metal cation activates it for attack by weak nucleophiles such as water. In this work, Cu^2+^ ions induce the ring-opening of epoxide groups on the graphene oxide sheets (Fig. 2(c)). During the process of mixing GO and Cu(Ac)_2_ solution, water as nucleophile attacks the coordination of the epoxy O atom to Cu^2+^ ion and then the epoxy group transform into a structure containing hydroxyl group and the coordination of hydroxyl group and Cu^2+^. Consequently, both the coordination between Cu^2+^ ions and carboxyl groups and the ring-opening of epoxy groups take place after Cu^2+^ ions are added, which supports that chemical interactions exist between GO and Cu^2+^ ions during MLM process.

In order to determine the reduction extent of graphene oxide in the GO-CuO composite particles, XPS was employed to analyze the GO-CuO composite powders and RGO-Cu composites. Curve fitting of the C1s spectra was performing using a Gaussian-Lorentzian peak shape after performing a linear background correction. Figure 2(c) shows the fitted peaks of sp^2^ hybridized carbon (284.6 eV)^35^, epoxy carbon(C-O, 286.3 eV) and carboxylate carbon (O-C = O, 288.9 eV)^36^. After reduction the relative intensities of C-O and O-C = O decrease obviously and the relative amount of sp^2^ carbon increases from 0.755 (GO-CuO) to 0.894 (RGO-Cu), which indicates that C-O and O-C = O groups are eliminated by the hydrogen reduction and that GO-CuO is mostly reduced into RGO-Cu by the hydrogen reduction.

### Preparation strategy

**Figure 3:**
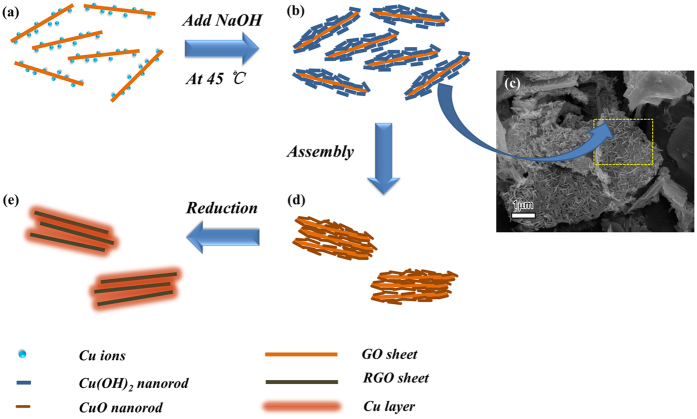
Schematic process of the production of the composite powder: (**a**) GO sheets in Cu(Ac)_2_ solution, (**b**) GO-Cu(OH)_2_ composite sheets, (**c**) SEM image of 2.5% composite powder (freezed dried), (**d**) assembled composite powder with micro-layered structure and (**e**) reduced composite powder.

A schematic diagram of the preparation process is shown in Fig. 3. First, GO and copper acetate are homogeneously mixed in deionized water by a rotor-stator mixer. As it was indicated that the zeta potential of GO with functional groups such as hydroxyl, epoxide, carbonyl, and carboxyl groups^3^ is between −40~−50 mV at the pH value of 6^37^. The GO surface with negative charges could attract Cu^2+^ in the solution and chemical bonds could be formed between the functional groups of the GO and the Cu^2+^, which has been proved in the FTIR analysis.

Secondly, Cu(OH)_2_ nanorods are *in-situ* produced on the surface of GO sheets when NaOH solution is dropped into the mixture solution within 1.5 h at 45 °C. The SEM image of the freeze-dried composite powder is displayed in Fig. 3(c). It can be found that the micro-sized GO sheets serve as excellent supporters^38^, and many nanorods are *in-situ* produced flattened on the surface of GO sheets forming a roughly plane structure as shown in Fig. 3(c). These nanorods should be Cu(OH)_2_ according to the analysis of XRD and ref. 38. It should be noted that the plane structure composed of nanorods on the surface of GO are not found in the process of MLM before, which may play an important role on the assembly of the micro-layered structure.

Thirdly, it is no doubt that van der Waals force and hydrogen bonding exist between the composite sheets shown in Fig. 3(b), they attract each other and assemble themselves into micro-layered structure^39^ during the mixing and centrifugation process as shown in Fig. 3(d). Further, the micro-layered structure can be retained during reduction treatment as demonstrated in Fig. 3(e).

### Microstructure characterization of bulk composite

**Figure 4:**
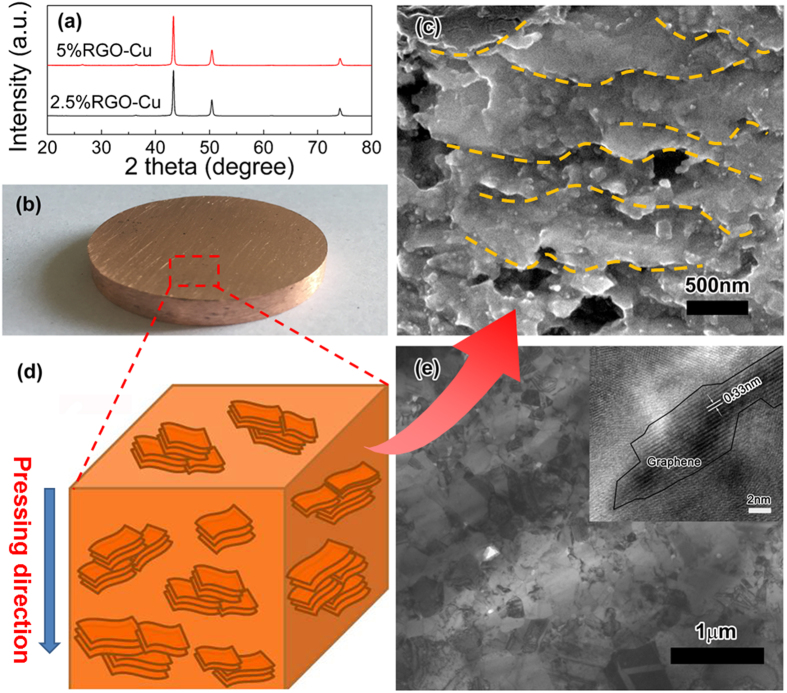
Microstructure characterization for bulk composite. (**a**) XRD patterns of 2.5 vol.% and 5 vol.% RGO-Cu composites (**b**) Photograph of RGO-Cu composite, (**c**) SEM image of deeply etched cross section of 5 vol.% RGO-Cu composite, (**d**) schematic model of RGO-Cu composite, and (**e**) TEM image of 5 vol.% RGO-Cu composite and the inset is a HRTEM image of the composite.

Figure 4(a) shows the XRD patterns of 2.5 vol.% and 5 vol.% RGO-Cu composite sintered by SPS process. The composites both exhibit only three characteristic peaks corresponding to Cu (PDF No. 851326), suggesting that copper matrix don’t be oxidized during the SPS process. In addition, the grain sizes of the Cu matrix for the samples can be estimated by Scherrer Formula. The Full Width Half Maximum (FWHM) of Cu peak at 74.1 degree for 2.5% RGO-Cu and 5% RGO-Cu composites are 0.281 and 0.316 degree, respectively. So the grain sizes of the Cu matrix are 34.1 nm and 30.7 nm according to Scherrer Formula. Therefore, the grain size may be a factor that affects the strength of composites but not the critical one. Sintered RGO-Cu composite is shown in Fig. 4(b), demonstrating a compact structure. Figure 4(c) is the SEM image showing the cross section of the deeply etched composite. The edges of RGO (highlighted by dotted lines) can be easily observed and almost parallel distributed; these RGO sheets can be considered as the framework of micro-layered grains. It is worth mentioning that Cu covered by graphene sheets cannot be easily etched because graphene has outstanding chemical resistance and blocks the flow of the etching solution^40^. It can be found that the composite consists of plenty of RGO-Cu micro-layered grains which tend to be perpendicular to the pressing direction as shown in Fig. 4(d), indicating that the composite powders rotate and adjust perpendicular to the pressing direction during the pressing sintering process.

Transmission electron microscopy analysis was further used to study the microstructure of RGO-Cu composites. As shown in Fig. 4(e), the grain size of Cu in the composites is in the range of several tens of nanometers to several hundred nanometers. These nano-sized grains should contribute to the strengthening of composites by fine crystal reinforcing mechanism. Furthermore, high-resolution TEM (HR-TEM) revealed the characteristic lattice fringes of graphene. And the interlayer space is 0.33 nm assigned to the (111) plane of graphene^41^. In addition, there was no pore or crack in the interface between Cu matrix and graphene sheet suggesting a well interfacial bonding.

### Mechanical and electrical properties

**Figure 5:**
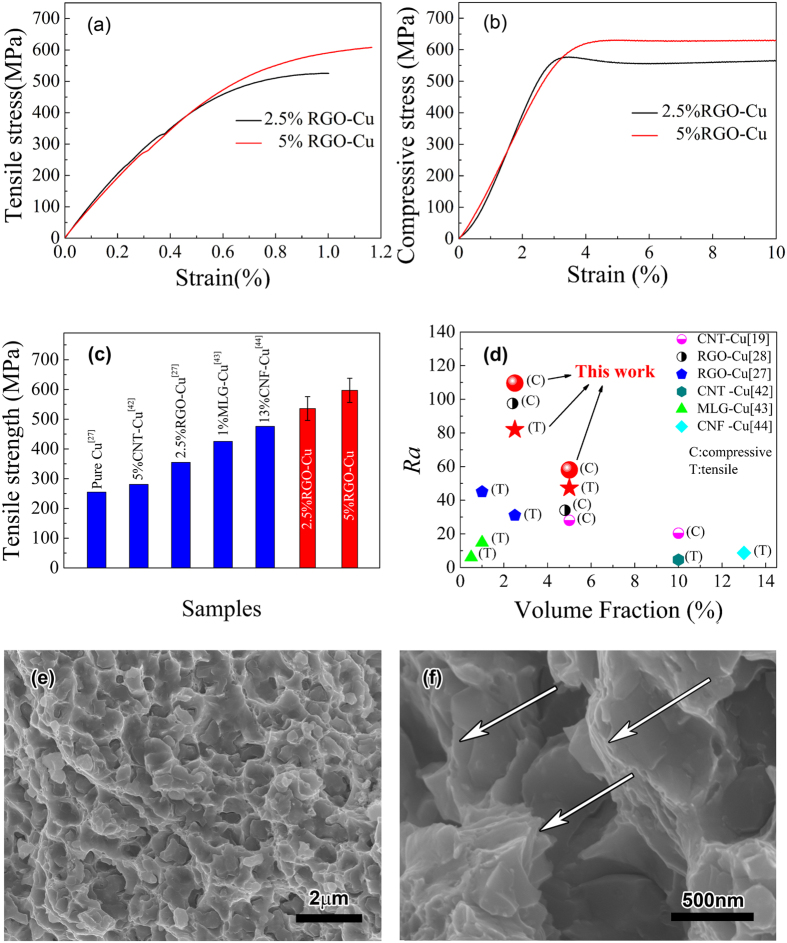
Mechanical properties of the bulk composite. **(a)** Tensile stress-stain curves of RGO-Cu composites, **(b)** compressive stress-stain curves of RGO-Cu composites, **(c)** tensile strength comparison of the composite of this work with those of pure Cu^27^, CNT-Cu composites^42^, RGO-Cu composite^27^, multi-layer graphene(MLG)-Cu composite^43^ and carbon nanofiber (CNF)-Cu composite^44^, **(d)** apparent strengthening efficiencies of several reinforcements in copper matrix composites derived from the data in previous studies and that in this work (RGO^27^,^28^, MLG^43^, CNF^44^ and CNT^19^,^42^), **(e)** and **(f)** fractographs of 5 vol.% RGO-Cu composite.

Tensile and compressive properties of RGO-Cu composites are shown in Fig. 5(a and b). The ultimate tensile strength of 2.5 vol.% RGO-Cu and 5 vol.% RGO-Cu composites are 524 and 608 MPa, respectively. As shown in Fig. 5(a), there is a gradual transition between elastic and plastic deformation, which suggests strain hardening occurs at the initial stage of plastic deformation. The obvious strain hardening in composites may be interpreted in terms of glide dislocation interaction with the interface between graphene and Cu matrix. Specifically, dislocations generate in Cu matrix and glide to the interface between graphene and Cu matrix. As the reinforcement, graphene can hinder the dislocation motion further due to its high strength and elastic modulus, which leads to dislocation pile-up at the interface. As a result of the dislocation pile-up at the interface, composites exhibit strain hardening.

The compressive strength of 2.5 vol.% RGO-Cu and 5 vol.% RGO-Cu composites are 576 and 630 MPa, respectively. It should be noted that the tensile strength of RGO-Cu composites in our method are almost equal to the compressive strength, which is seldom mentioned in other graphene-Cu matrix composites. Since the tensile strength is sensitive to macro defects, our result means that the composites have an uniform structure with less macro defects such as cracks and holds.

The tensile strength of pure Cu, RGO, CNT, MLG and CNF reinforcing Cu-matrix composites are shown in Fig. 5(c). It is striking to note that the maximum tensile strength of 5 vol.% RGO-Cu composite in our method (608 MPa) is 2.4 times greater than that of pure Cu (255 MPa)^27^, which is the highest tensile strength of Cu-matrix composite materials at centimeter level reinforced by nano carbon fillers so far. Moreover, the tensile strength of 2.5 vol.% RGO-Cu composite prepared using our method is much higher than that of RGO-Cu composite prepared using conventional MLM method^27^ with the same volume fraction. Furthermore, the tensile strength of 5 vol.% RGO-Cu composite is higher than that of 2.5 vol.% RGO-Cu composite suggesting that the volume fraction of RGO in our composites can reach to 5 vol.% without the obvious agglomeration of GO sheets, higher mechanical properties can also be expected by increase the volume fraction of the reinforcements.

Figure 5(d) shows a comparison of the apparent strengthening efficiencies (*Ra*) of several reinforcements in Cu matrix with their volume fraction. The apparent strengthening efficiency can be expressed as:


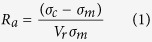


where *σ*_*c*_ is the yield strength of the composites, *σ*_*m*_ is the yield strength of the matrix, *V*_*r*_ is the volume fraction of the reinforcement^28^. In this work, σ_m_(C) and *σ*_*m*_(T) are defined as 150 MPa from ref. 19 and 160 MPa from ref. 27 for easier comparison; the letter C in the bracket stands for compressive strengthening, while the letter T stands for tensile strengthening. In the Fig. 5(d), the tensile and compressive strengthening efficiency of RGO in this work reaches to 82 and 110, respectively, which is considerably higher than that of CNT^42^, MLG^43^, CNF^44^ and RGO^27^ in the conventional MLM method. It manifests that the RGO-Cu composite with micro-layered structure exhibits excellent strength with low volume fraction. In addition, it should be noted that the composites mentioned in ref. 28, which also uses the MLM method and introduces high-shear mixing to improve the dispersity of graphene, has lower apparent strengthening efficiency (97.5) than those produced in this study with the same volume fraction.

As shown in Fig. 5(e and f), we can see that the structure of the fracture surface of RGO-Cu composite is homogeneous and the micro-layered structure that is obvious on the detail view of the fracture surfaces (marked by arrows). And dimples and tear ridges of Cu matrix can be observed from Fig. 5(e). It indicates that the plastic deformation has occurred in the Cu matrix before fracture, as shown in tensile stress-strain curves (Fig. 5(a)). Thus Cu matrix acts as the main source of plasticity in composites. In addition, planes and cracks can be also observed as shown in Fig. 5(f), suggesting that the interfacial strength between graphene and copper is not high enough. As mentioned above, dislocations pile up at the interface between graphene and copper, resulting in interfacial crack ultimately. Therefore the interface between graphene and Cu matrix is still the weak link in composite, which is worth studying further.

The hardness values of RGO-Cu composites measured by Vickers hardness are shown in Table 1. The hardness of 5 vol.% RGO-Cu in this work is as high as 188.8 HV, which is almost 3 times of pure copper (63 HV)^45^ and is about 10% higher than that of 2.5 vol.% RGO-Cu. It is worth noting that the hardness of RGO-Cu composite prepared by the conventional MLM method is 109 HV, which is only about 65% of that of RGO-Cu with the same volume fraction of graphene. This is because the interaction between graphene reinforcement and copper matrix is greatly weakened at the high temperature. These results show that the hardness of RGO-Cu composites has similar trend with the compressive strength.

Furthermore, the electrical conductivity of RGO-Cu composites has been measured and expressed as the International Annealed Copper Standard (IACS). The conductivity value of 2.5 vol.% RGO-Cu in this study is 65.5% IACS, while the composite with the same volume in MLM method has much lower conductivity (53.2% IACS)^46^. And 5 vol.% RGO-Cu maintains a good electrical conductivity property as high as 62.0% IACS.

As a result, the excellent tensile and compressive strengths, high hardness and good electrical conductivity are obtained simultaneously in the RGO-Cu composites. The excellent properties of the composites are closely related with the unique layered structure produced during the MLM process. Previous reports on metal layered systems^47^ has proved that graphene with a high strength can provide an efficient barrier to the dislocation motion across the interface between graphene and metal^25^. Moreover, the unique micro-layered structure makes Cu matrix isolated by graphene sheets and lowers the grain size of the copper matrix, which is in the range of several tens of nanometers to several hundred nanometers; so fine grain strengthening also plays a role in the excellent strength of the composite. Consequently, our work is an active exploration to produce composite with layered structure and excellent properties in a relatively large scale.

At the end of the paper, a question should be answered why the composites in this work can produce an obvious micro-layered structure but not the other composites with similar MLM processes. In our opinion, the main reason may be the reaction temperature of the MLM process. The reaction temperature in this work is 45 °C while it is generally 80 °C in other works. Our previous work shows that CuO particles are produced by heating cuprammonia solution at 80 °C, which don’t go through the Cu(OH)_2_ nanorod step. However, with the decrease of the reaction temperature to 45 °C, Cu(OH)_2_ nanorods can be *in-situ* produced on the surface of GO and then assemble into the micro-layered structure. So the reaction temperature plays an important role in the formation of micro-layered structure. Another interesting question is what should happen if the reaction temperature decreases further or the other factors in the MLM process change, it is no doubt that further work should be done to clarify the main factors controlling the structure of copper compounds and further explore the potential of the composite properties.

## Conclusions

In summary, RGO-Cu composites with micro-layered structure are fabricated based on MLM method. The composite contains many micro-layered grains consisting of alternating layers of graphene and copper. Cu(OH)_2_ nanorods *in-situ* produced on the surface of GO play an important role on the formation of the micro-layered structure, which has significant influence on the property of the RGO-Cu composites. The excellent tensile and compressive strengths, high hardness and good electrical conductivity are obtained simultaneously in the RGO-Cu composites. The remarkable reinforce effect of graphene in our composites derives from the RGO-Cu micro-layered structure that enhances the uniform dispersion of graphene and prevent slipping between graphene flakes. The results shown in the present study provide an effective method to design graphene based composites with layered structure and high performance. Further work should be done to explore the potential of the composite properties by further clarifying the main factors in the process of MLM.

## Methods

### Fabrication of GO

Graphite oxide was made by a modified Hummers method^48^. Graphite (1 g), H_2_SO_4_ (45 ml) and H_3_PO_4_ (5 ml) were mixed in an ice water bath. KMnO_4_ (7 g) was added slowly as an oxidizing agent into the mixture solution under fast stirring and the reaction maintained below 20 °C for 30 min. The solution was then heated slowly to 50 °C and stirred for 10 h. After cooled to room temperature and the solution was diluted by deionized water in the ice water bath and poured out. Then appropriate H_2_O_2_ was added to remove redundant KMnO_4_ until the solution became golden yellow and the bubbles didn’t appear anymore. Finally, it was rinsed by 10 vol% HCl solution (2000 ml) to remove Mn ions and rinsed by deionized water to remove Cl^−^ ions.

### Fabrication of GO-CuO and RGO-Cu composite powders with micro-layered structure

Graphite oxide suspension (1 mg/ml, 500 ml) was treated by sonicating with a KQ-800KDE ultrasonic cleaner (Kun Shan Ultrasonic Instruments Co., Ltd) for 2 h; then a brown yellow graphene oxide colloid was formed. 112 g Cu(CH_3_COO)_2_ (Cu(Ac)_2_) was added into deionized water for the preparation of 2000 ml aqueous solution and then mixed with 500 ml GO colloid for 30 min by a rotor-stator mixer (JRJ300-1) with the rotating speed of 3000 rpm, which provided vigorous stirring to mix uniformly. Then the solution was gradually heated to 45 °C and NaOH aqueous solution (4 M, 500 ml) was added drop by drop. The suspension was allowed to stir for another 20 min. Then the composite powders were separated from the solution by centrifugation and rinsed with deionized water until the cleaning liquid was neutral. Next, the slurry was dried in vacuum at 110 °C for 4 h. Further, composite powders were reduced at 400 °C for 5 h under the atmosphere of H_2_-Ar mixed gas (H_2_ content: 17%) with a gas flow rate of 100 sccm. Finally, the RGO-Cu composite powders were produced.

### Consolidation of the RGO-Cu composite particles

The RGO-Cu composite particles were sintered by spark plasma sintering (SPS, SPS-1050) at 600 °C for 5 min with an applied pressure of 40 MPa, a vacuum of 0.1 Pa and a heating rate of 75 °C/min. The size of the compacted RGO-Cu composite was 30 mm in diameter and 5 mm in thickness.

### Characterization of the RGO-Cu composite

X Ray Diffraction (XRD) was carried on a Philips X’Pert X-ray diffractometer with Cu K_α_ radiation (λ = 1.54 Ǻ) for phase analysis. Raman spectra were preformed from 500 to 3000 cm^−1^ on a B&W Tek Confocal Micro-Raman spectrometer using a 532 nm laser. Fourier Transform Infrared spectra were recorded on a Nicolet Avatar-360 spectrometer using KBr powder pressed pellets. X-ray photoelectron spectroscopy (XPS) was obtained by Thermo Fisher). The surface of samples was etched to exclude the influence of surface oxidation and adventitious carbon. All XPS peaks are calibrated according to the C 1 s peak (284.6 eV).

The microstructures of RGO-Cu composite particles and composite materials were observed by an optical microscope (OM, OLYMPAS PMG3), scanning electron microscopy (SEM, Helios Nanolab600i) and transmission electron microscope (TEM, TecnaiF2F30).

Tensile and compressive tests were performed using an electronic universal testing machine (Instron-5569) with a crosshead speed of 0.5 mm/min. The tensile specimens were 30 mm in length, 2 mm in width and 1 mm in thickness. The loading directions in tensile tests are perpendicular to the processing direction of the samples. And the compressive specimens had a cylindrical shape with 5 mm in height and 3 mm in diameter. The loading directions in compressive and tensile tests are paralleled and perpendicular to the processing direction of the samples. The electronic resistivity and hardness of composites were tested by direct current low resistance test instrument from Suzhou Changsheng Technology Co. and digital micro vickers hardness tester (HXD-1000TM/LCD). The test directions are perpendicular to the processing direction of the samples.

## Additional Information

**How to cite this article:** Wang, L. *et al*. Graphene-copper composite with micro-layered grains and ultrahigh strength. *Sci. Rep.* **7**, 41896; doi: 10.1038/srep41896 (2017).

**Publisher's note:** Springer Nature remains neutral with regard to jurisdictional claims in published maps and institutional affiliations.

## Figures and Tables

**Table 1:** The electrical conductivity and hardness of RGO-Cu composites.

Sample	Hardness (HV)	Conductivity (%IACS)
2.5 vol.% RGO-Cu (This work)	161.7	65.5
5 vol.% RGO-Cu (This work)	188.8	62.0
2.5 vol.% RGO-Cu^47^	105	53.2
Pure Cu^46^	63	—
